# Supplementary material for: Experiences of a mobile phone delivered brief intervention for hazardous drinking: A qualitative study nested in the AMBIT trial from Goa, India
Source: Glob Ment Health (Camb). 2023 Sep 7;10:e58. doi: 10.1017/gmh.2023.51 (PMC10579675; doi:10.1017/gmh.2023.51)
Supplement: Fernandes et al. supplementary material 1 — Fernandes et al. supplementary material [file S2054425123000511sup001.docx]

Appendix 1

INTRODUCTION

Good morning/Good afternoon! Thank you for meeting with me today.

You have been part of a mobile-based intervention to improve risky drinking patterns, for the past 8 weeks. During these weeks you have received messages to help you manage your drinking. We are meeting with you today to understand your experience of this intervention and get feedback on improving the delivery of this intervention here in Goa.

Over the course of the intervention, you may have found some of the messages helpful or some messages may have been unhelpful to you. You may have liked receiving some messages or disliked some messages or parts of the intervention. All these perspectives are valid and will help us to improve this intervention. Could you share your opinion of the overall experience with this intervention? If you are ready, I will go ahead and turn on the recorder now. Shall we begin? Let me start by asking you some questions about your overall experience.

OBJECTIVES

1. What changes in alcohol use/ behaviour at the end of the intervention?
2. What are the mechanisms that created this change?
3. What was the experience of receiving health service over phone? What worked well? /What barriers? what would be better?
4. Was the delivery convenient and feasible? How did this affect participation? /what would be better?

GENERAL

Thank you for agreeing to participate in this intervention.

1. During the program you received messages and sometimes you were asked a question and had to respond. I would like to know your experience with responding to messages.

Probes:

- How do you feel about responding to our messages? Why?/ How did this affect participation?/ Can you tell me more about this?
- How do you feel about receiving information without any response required from your side? Why?/ How did this affect participation?/ Can you tell me more about this?

1. How do you feel about receiving information through SMS or IVR?

Probes:

- How did interacting with SMS or IVR affect participation?
- What mode of delivery would be ideal to receive this information?
- Why is this mode preferable? / Can you tell me more about this?

1. What challenges did you face that prevented participation in the program?

PERCEIVED IMPACT

1. During this 2-month intervention what changes have you experienced in your drinking pattern?

Probes-

- Tell me about the overall change/ How did it change?
- Why do you think this change occurred?
- How has your consumption changed? How has your frequency of drinking changed?

1. How has the program affected the way you think about drinking?

Probes-

- Tell me about these changes. / How has the way you think about drinking changed?
- Can you tell me about information you have learnt about managing your drinking?
- Can you tell me how you have used this information in your daily life?

CLOSING

1. How could we improve the intervention?

OBJECTIVES (To Check)

1. What changes in alcohol use/ behaviour at the end of the intervention?
2. What are the mechanisms that created this change?
3. What was the experience of receiving health service over phone? What worked well? /What barriers? what would be better?

Thank you very much for your time. Your feedback and suggestions are a valuable contribution to help us to develop this intervention.
